# Morphine-induced supraventricular tachycardia in near-term fetus

**DOI:** 10.1186/s13052-018-0570-1

**Published:** 2018-09-24

**Authors:** Zanardo Vincenzo, Simbi Alphonse, Parotto Matteo, Severino Lorenzo, Carta Riccardo, Guerrini Pietro, Straface Gianluca

**Affiliations:** 1 Division of Perinatal Medicine, Policlinico Abano Terme, Piazza Colombo 1, 35031 Abano Terme, Italy; 2 Department of Anesthesia, University of Toronto, Toronto, ON Canada

**Keywords:** Fetal supraventricular tachycardia, Morphine, Cesarean delivery

## Abstract

**Background:**

Fetal supraventricular tachycardia (SVT), characterized by fetal heart rate between 220 and 260 bpm, is a rare but most commonly encountered fetal cardiac arrhythmia in pregnancy that may be associated with adverse perinatal outcome.

**Case presentation:**

We describe a 36/6 week near term fetus who presented morphine-induced SVT after maternal treatment of a renal colic. Following emergency cesarean section, the neonate had resolution of symptoms.

**Conclusions:**

The pathophysiology of morphine-related SVT, previously documented in experimental animal models, and for the first time reported in the human fetus, is presented.

## Background

Fetal tachycardia, first recognized in 1930 by Hyman et al., is a condition occurring in approximately 0.4–0.6% of all pregnancies [1, 2]. Fetal supraventricular tachycardia (SVT), characterized by fetal heart rate (HR) between 220 and 260 beats per minute (bpm) is a rare but most commonly encountered fetal cardiac arrhythmia in pregnancy that may be associated with adverse perinatal outcome [3, 4]. A subset of these cases with more sustained periods of tachycardia is clinically relevant for enhanced risk of cardiac failure, non-immune hydrops, and fetal death [5]. Excessive caffeine, smoking, illicit drugs (i.e. cocaine) exposure, hyperthyroidism in pregnant women and cardiac malformations or extracardiac malformations (i.e. diaphragmatic hernia) in fetuses, respectively may contribute to frequent fetal premature atrial contractions which may progress to unrelenting tachyarrhythmia [6]. If no underlying cardiac defects are present, medical management may be used with equivocal success, leading to levels of anxiety among pregnant women and treating physicians [3].

In this report, a case of morphine-induced SVT in near-term fetus after maternal treatment of a renal colic, is presented. The pathophysiology of morphine-related rhythm disturbances, previously documented in experimental animal models [7, 8] and for the first time reported in the human fetus, and a review of pertinent literature are also presented.

## Case presentation

A 38-year-old Gravida 3 lady at 36/6 weeks of gestation presented to our labor and delivery unit for a renal colic. Her prenatal course was unremarkable. She had no past surgical or medical history, but she was presenting acute pelvic pain, related to renal colic. Her vital signs were as follows: blood pressure 115/68 mmHg, HR 73 bpm, respiratory rate 17 breaths per minute, and temperature 36.7 °C. Her physical exam was unremarkable. Maternal baseline ECG was normal. Baseline electrolytes and maternal thyroid panel were within normal limits. There was absense of fever and of blood and urine infection indexes. After ruling out threatening labor, fetal ultrasound showed grossly normal fetus with an estimated weight of 2,783 g and amniotic fluid index 14.6 cm. The anatomical survey including fetal cardiac evaluation was found to be within normal limits. The 4-chamber view of the heart was normal. No signs of hydrops fetalis were noted. Electronic fetal HR monitoring showed a sustained baseline fetal heart of 140 bpm with minimal to moderate variability, with absent accelerations or decelerations and not relevant uterine contractile activity. Middle cerebral artery Doppler ultrasonography was also within normal.

**Fig. 1:**
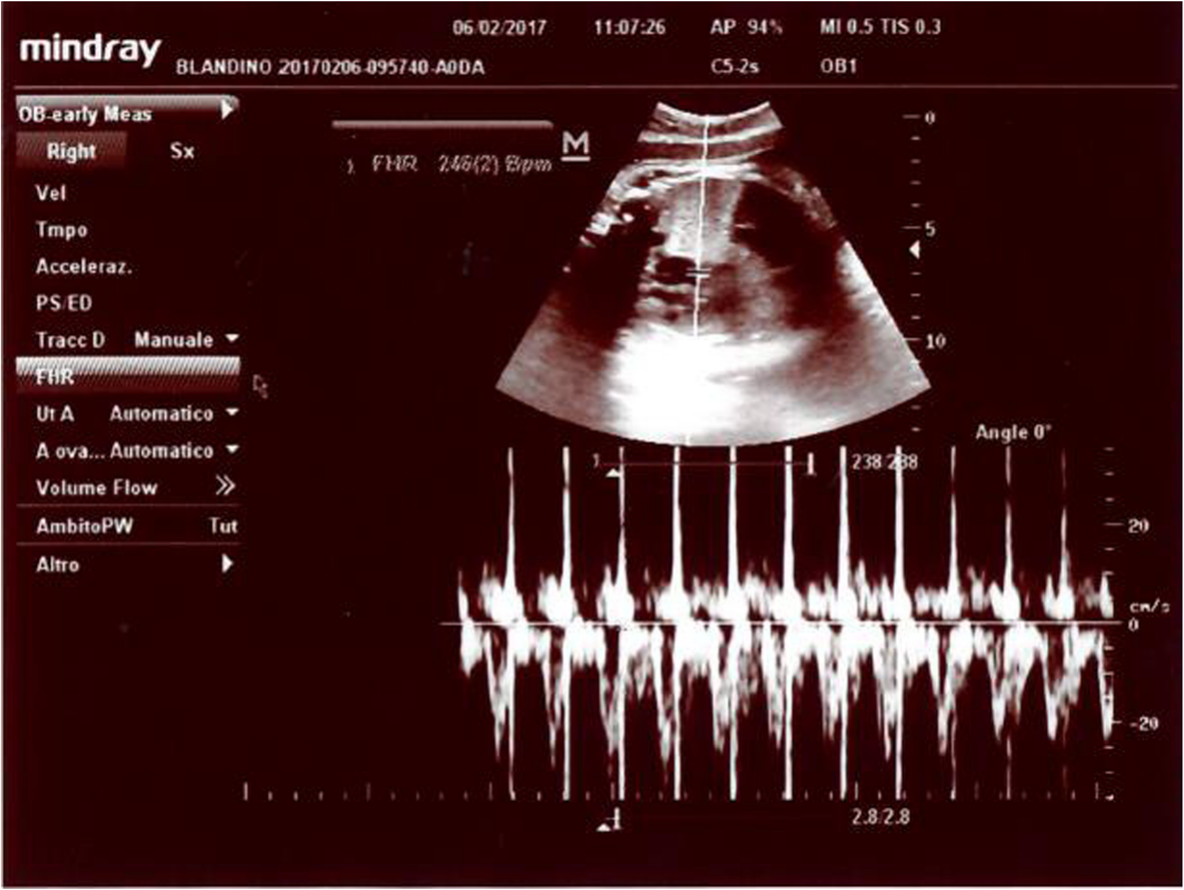
Simultaneous pulse Doppler recording obtained via five-chamber view. The heart rate was 246/min

Spasmolytic therapy (Butylscopolamine) did not resolve colic pain. The following day, although her physical exam was unchanged, a plan was made for administration of betamethasone series for lung maturity and morphine for colic pain control. Immediately after morphine intravenous administration (0.1 mg/kg), electronic fetal HR monitoring showed a sudden sustained increase of baseline fetal heart from 140 bpm to over the upper range limits cardiotocography (CTG) registration (200 bpm). Fetal echo on M mode revealed a 1:1 atrial ventricular rate of 240 bpm consistent with SVT (Fig. 1). Conversely, heart rate, blood pressure, respiratory rate, and temperature of the mother were substantially unchanged.

After 4 h fetal HR was sustained at 240 bpm. Since the fetus was at a reasonable maturity stage, immediate delivery was decided. A baby boy was delivered following a caesarean section, with a 2920 g birth weight, APGARs of 10 and 10 at 1 and 5 min, umbilical artery cord blood pH 7.37, BE-1.7. He was admitted to the regular nursery. Pre-ductal saturation was 99%. Mean blood pressure 38 mmHg. Tachycardia was noticed at birth 240 bpm, and the HR progressively decreased starting at 60 min after birth and reached normal levels during the following 3 h.

Postnatal adaptation was uneventful and exclusive breastfeeding was carried on. After a 2-days period of neonatal cardiac monitoring (including echocardiography) the mother and her baby were discharged home in stable condition. The neonate progressed well. His weight increased normally and there was no evidence of cardiac rhythm disturbance up to the 1-year follow-up as outpatient in the pediatric cardiology department.

## Discussion

Fetal SVT is a rhythm disturbance characterized by sustained fetal heart rate between 220 and 260 beats per minute. With the rapid advancement in fetal echocardiographic techniques, sustained SVT with a fetal heart rate (FHR) more than 220 bpm can be diagnosed accurately during prenatal life [4].

We reported, for the first time, a case of morphine-induced SVT in a near-term fetus diagnosed by using M-mode and pulsed Doppler ultrasonography, in close temporal relationship with the mother treatment with opioid for a lasting painful renal colic. Since the fetus was at a reasonable maturity stage, with a normal anatomical survey at the time of diagnosis and without maternal and fetal known confounding factors which may progress to unrelenting tachyarrhythmia, immediate delivery by cesarean section was decided, and the baby was healthy.

The predominant mechanism of developing SVT in fetus is believed to be due to atrio-ventricular reentrant tachycardia [9, 10]. With advances in ultrasound technology it is now possible to diagnose accurately the type of arrhythmia using M-mode echocadiogram to compare the relationship between atrial and ventricular contractions. Fetal SVT is defined as 1:1 atrioventricular activity of the FHR exceeding 200 bpm. It accounts for 60–80% of the fetal tachyarrhythmias with a prevalence ranging from 1/1000 to 1/25 000 pregnancies [10]. The clinical presentation of fetal SVT has, however, a wide spectrum [4]. It can be intermittent with no hemodynamic effects to persistent type with high output cardiac failure leading to hydrops fetalis. Sustained fetal tachycardia is defined as fetal HR greater than 160 bpm, and presents in more than 50% of the time on fetal heart monitoring [11]. Initial evaluation of a fetus with arrhythmia should also include a detailed history and physical examination, particularly of congenital defects like diaphragmatic hernia contributing to frequent fetal premature atrial contractions, which may progress to unrelenting tachyarrhythmia, to guarantee an individualized management. There are confounding factors which can be maternal, such as maternal history of arrhythmias, caffeine consumption, smoking, and illicit drugs abuse (i.e. cocaine) [6].

According to the American Heart Association, in utero SVT management should be based on gestational age, presence and degree of fetal compromise, maternal condition, degree of tachycardia, intermittent versus sustained tachycardia, and whether hydrops is present or not [12, 13]. Unless near term, all pregnant women with sustained fetal tachycardia should receive pharmacological treatment. Management is primarily based on transplacental antiarrhythmics such as digoxin, sotalol, flecainide, amiodarone, or propranolol with the goal to slow ventricular rate and avoid hydrops [14–16]. Success of single drug therapy varies between 50 and 70%. In fact, in utero management of fetal SVT is challenging because transplacental drug transfer may decrease as a result of placental oedema, and no clear consensus exists regarding the best drug regimen for fetal SVT. Fetal response to therapy that may take several days after achieving maintenance dose [4]. In addition, pharmacological agents are prescribed on the basis of the arrhythmia itself, rather than on the precise knowledge of the location of the abnormal conduction pathways. Conversely, in the non-hydropic fetus in which fetal lung maturity can be demonstrated (usually > 34 weeks of gestation) delivery with evaluation of the neonate should be considered [17].

Pregnant patients with renal colic commonly present to the emergency department, and urolithiasis-associated pain is among the most agonizing visceral pain syndromes. Rapid and effective analgesia is a priority in renal colic, and intravenous nonsteroidal anti-inflammatory drugs and opioids are standard treatments [18]. Morphine is readily distributed to the fetus after maternal administration [19], but to the best of our knowledge, in utero fetal SVT after maternal morphine course in the treatment of a renal colic was never reported.

The role of the endogenous opioid system in the control of cardiovascular function has been a subject of research in adults for many years. In general, a suppressive action on heart rate has been reported. By contrast, in animal models, few fetal studies have focused on cardiovascular effects of opioids and opioid antagonists, making results difficult to compare and rather controversial. For example, in Zhu and Szeto study, administration of opiate agonists to the fetal lamb resulted in fetal sinus tachycardia, with HR increase in a dose dependent manner and in a rather unusual bell-shaped dose-response [7]. Conversely, bradycardia was not observed at any dose. Interestingly, the administration of naloxone to the fetal sheep caused an increase in fetal HR as well [20].

The mechanisms of action of morphine-induced tachycardia are also poorly understood. The study by Zhu and Szeto in fetal lambs [7] demonstrated that the cardio-acceleratory effect of morphine can be completely abolished by propranolol, suggesting that it is mediated via the β adrenergic system [21]. This observation is supported by experimental data in adult animals after morphine [22] and other opioid peptides administration [23]. Morphine and other opioid peptides have been shown to increase both brain and plasma levels of epinephrine, norepinephrine and dopamine [24]. There is evidence that the opiates increase central sympathetic outflow to the adrenal medulla. Our present case report is consistent with these reasonable hypotheses.

## Conclusion

In conclusion, the present report suggests that morphine can produce SVT in the near-term human fetus, similarly to what was described in the anaesthetized sheep fetus. It is probably a specific opiate-receptor action and is mediated, at least in part, by activation of beta-adrenergic pathways. Further studies are needed to explore the exact mechanism and sites of morphine, opioid antagonists action, and the role beta-blockers on human fetal cardiovascular function.

## Abbreviations

Bpm: Beats per minute
HR: Heart rate
SVT: Supraventricular tachycardia
