# Correction to: Morphine-induced supraventricular tachycardia in near-term fetus

**DOI:** 10.1186/s13052-019-0612-3

**Published:** 2019-02-04

**Authors:** Vincenzo Zanardo, Alphonse Simbi, Matteo Parotto, Lorenzo Severino, Riccardo Carta, Pietro Guerrini, Gianluca Straface

**Affiliations:** 1 Division of Perinatal Medicine, Policlinico Abano Terme, Piazza Colombo 1, 35031 Abano Terme, Italy; 2 Department of Anesthesia, University of Toronto, Toronto, ON Canada

**Correction to: Ital J Pediatr**

**https://doi.org/10.1186/s13052-018-0570-1**

The original article [1] contained an error whereby all authors’ names were mistakenly inverted. This has now been corrected.
